# Efficiently Computing Excitations of Complex Systems: Linear-Scaling Time-Dependent Embedded Mean-Field Theory in Implicit Solvent

**DOI:** 10.1021/acs.jctc.1c01133

**Published:** 2022-02-08

**Authors:** Joseph C. A. Prentice

**Affiliations:** Department of Materials, University of Oxford, Parks Road, Oxford OX1 3PH, United Kingdom

## Abstract

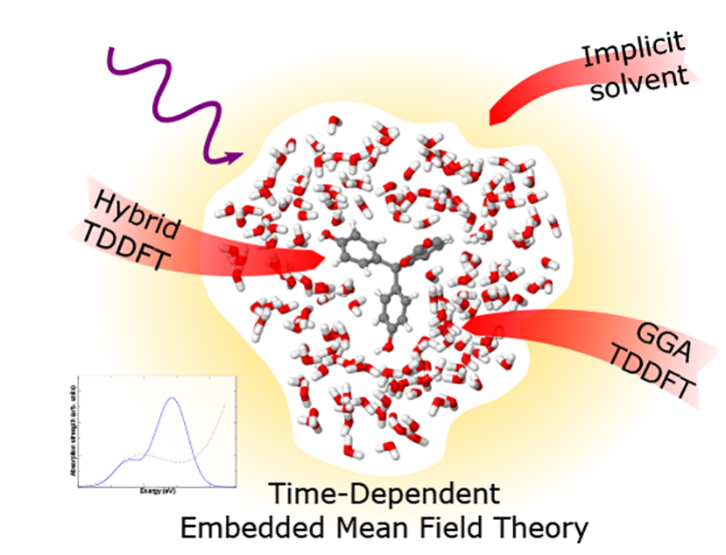

Quantum embedding schemes have the potential to significantly reduce the computational cost of first-principles calculations while maintaining accuracy, particularly for calculations of electronic excitations in complex systems. In this work, I combine time-dependent embedded mean field theory (TD-EMFT) with linear-scaling density functional theory and implicit solvation models, extending previous work within the ONETEP code. This provides a way to perform multilevel calculations of electronic excitations on very large systems, where long-range environmental effects, both quantum and classical in nature, are important. I demonstrate the power of this method by performing simulations on a variety of systems, including a molecular dimer, a chromophore in solution, and a doped molecular crystal. This work paves the way for high accuracy calculations to be performed on large-scale systems that were previously beyond the reach of quantum embedding schemes.

## 1 Introduction

Embedding schemes are a well-studied method for improving the computational efficiency of calculations on complex systems without significantly sacrificing accuracy. These schemes are best suited to systems where the relevant physics is dominated by a small “active” subregion, but the rest of the system still affects this behavior on an environmental level.^1^ In such systems, a certain level of theory may be required to accurately describe the relevant physics, but applying this level of theory to the whole system is often infeasibly computationally expensive. Examples could include molecules in solution,^2−4^ host–guest systems,^5−7^ defects in crystals,^8−10^ and active sites in enzymes.^11−13^ Embedding schemes seek to solve this problem by treating the active region with an accurate, but computationally intensive, “higher” level of theory, while the environment is treated with a less demanding, but less accurate, “lower” level of theory. Using the higher level of theory for the active region only means that the most important contributions to the property under study are still described accurately, while using the lower level of theory for the rest of the system reduces the computational cost but still allows the environment to influence the result.

Embedding schemes can be divided into those that treat the environment classically^14,15^ and those that treat the environment quantum mechanically,^16−22^ allowing for quantum mechanical interactions between the regions;^1^ the latter class are known as quantum embedding schemes. One recently proposed such scheme is embedded mean-field theory (EMFT).^23^ One of the advantages of EMFT over other quantum embedding schemes is that it is a mean-field theory, like density functional theory (DFT), so many existing methods that have been built on the foundations of DFT can be easily modified to accommodate EMFT. EMFT has been successfully used several times since its proposal,^24−29^ largely focused on relatively small molecular systems. In a previous publication,^30^ however, the author and co-workers extended the applicability of EMFT to large-scale periodic systems by presenting a novel combination of EMFT and linear-scaling DFT in the code ONETEP.^31^ This work demonstrated EMFT’s utility for hybrid DFT-in-semilocal DFT embedding calculations on large-scale systems, such as molecular crystals, but focused on calculating ground state energies only. Although we were able to access some excited state properties, studying the excited states of such large systems more generally with EMFT was not considered.

One of the most popular methods for calculating electronic excitations is time-dependent density functional theory (TDDFT). TDDFT is popular for its balance of reasonable accuracy and relatively low computational cost.^32,33^ However, standard semilocal TDDFT has several known issues, including its failure to correctly describe charge transfer states^33,34^ and the underestimation of excitation frequencies.^4^ These issues can be partially fixed by using hybrid functionals, including range-separated hybrids, but these are significantly more computationally expensive.^34^ Quantum embedding offers a way to obtain the accuracy of these methods, while significantly lowering the computational cost. The combination of linear-response TDDFT and EMFT (known as TD-EMFT) has previously been implemented and found to work well, but has only been applied to small molecular systems.^26^

In this work, I extend the previously described novel combination of EMFT and linear-scaling DFT to include TD-EMFT, allowing electronic excitations to be computed using this scheme. I also combine this implementation with the implicit solvation model present in ONETEP,^31,35^ allowing for both EMFT and TD-EMFT calculations to be placed in a continuous dielectric medium with a given permittivity. This makes multilevel calculations of electronic excitations possible, for example, using a hybrid functional to describe the active region, a semilocal function to describe the nearby environment at a quantum level, and then implicit solvent to describe the rest of the environment at a continuum level. This allows for computationally efficient and highly accurate TDDFT calculations to be performed on much larger systems than would previously have been possible. I have tested this implementation on a range of different systems, demonstrating the breadth of potential applications.

The work is organized as follows. In section 2, I give a brief overview of the theory of (TD-)EMFT as described in previous work and how this is implemented in ONETEP. In section 3, I give the results of testing our linear-scaling TD-EMFT implementation on several systems: a water-nitrogen dimer (section 3.1), phenolphthalein solvated in water (section 3.2), and a pentacene-doped *p*-terphenyl molecular crystal (section 3.3). Finally, in section 4, I give some concluding remarks.

## 2 Background Theory

In this work, atom-centered basis functions are used, which in general will be nonorthogonal. Because of this, the overlap matrix *S*, which gives the overlaps between basis functions, acts as a metric tensor in the space spanned by the basis functions. As *S* is not simply the identity in general, a distinction must be drawn between covariant and contravariant quantities, represented with subscript and superscript indices in the following. A contravariant quantity χ^β^ can be transformed into its dual covariant quantity χ_α_ by applying *S*: χ_α_ = ∑_β_*S*_αβ_χ^β^, where α, β run over basis functions. Conversely, covariant quantities can be transformed into their dual contravariant quantities using the inverse overlap *S*^–1^: χ^β^ = ∑_α_(*S*^–1^)^βα^χ_α_. Greek indices are used to enumerate the basis functions, with capital Latin indices representing different embedding regions.

### 2.1 Ground-State Embedded Mean-Field Theory

As outlined in previous work, EMFT is based on splitting the system into two regions, the active region A and the environment B, at the basis set level.^23^ For atom-centered basis sets, this simply means assigning each atom to a particular region, which then assigns all basis functions associated with that atom to that region too. If the density matrix is expressed in terms of these basis functions (also known as the density kernel *K*(31)), it can be separated into blocks corresponding to the regions:
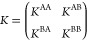
1

A similar expression applies for the overlap matrix *S*. The density of the full system can then be calculated as ρ(**r**) = ∑_αβ_ϕ_α_^*^(**r**)*K*^αβ^ϕ_β_(**r**), where ϕ_α_(**r**) are the basis functions. Densities corresponding to the various blocks of *K* can be calculated as ρ_*IJ*_(**r**) = ∑_α∈*I*,β∈*J*_ϕ_α_^*^(**r**)*K*^αβ^ϕ_β_(**r**).

The energy can now be written as a functional of *K* in its most general form for a mean-field theory, as EMFT is only applicable to mean-field theories. The energy is given by^23,30^
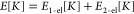
2where *E*_1-el_ corresponds to the energy arising from all one-electron terms in the Hamiltonian, and *E*_2-el_ corresponds to the energy arising from all two-electron terms. In DFT, *E*_1-el_ includes contributions such as the kinetic and electron–nuclear contributions to the energy, while *E*_2-el_ includes the Hartree and exchange-correlation contributions.

As this work focuses on DFT-in-DFT embedding, the higher and lower levels of theory can be assumed to differ only in the two-electron term; the higher level of theory would have *E*^high^[*K*] = *E*_1-el_[*K*] + *E*_2-el_^high^[*K*], while the lower level would have *E*^low^[*K*] = *E*_1-el_[*K*] + *E*_2-el_^low^[*K*]. The key assumption of EMFT is then that the energy can be written as^23,30^
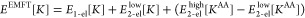
3

Three energy evaluations are required to evaluate this expression. First, the energy of the whole system (including the one-electron terms) is calculated at the lower level of theory. Next, the two-electron terms are computed twice using the *K*^AA^ sub-block of the density kernel only, once at the lower level of theory and once with the higher level. The difference of these two quantities is calculated and added on as a correction to the energy of the whole system calculated previously. All the quantities computed here are calculated at the mean-field level, so this is a mean-field theory.

In DFT-in-DFT embedding, *E*_2-el_ depends on the exchange-correlation functional chosen. The most logical choice for the lowest level of theory is to use a semilocal functional, with the higher level of theory using a more computationally demanding type of functional, such as a hybrid functional. Importantly, hybrid functionals include a fraction of exact exchange energy. Exact exchange, unlike the other energy terms discussed so far, is not a functional of the density, so it needs to be treated differently. The least computationally expensive way of calculating the exact exchange contribution to the energy of the active region is the EX0 method.^23^ This only includes exchange within the active region, neglecting exchange between the active region and the environment. It is possible to include exchange between the active region and the environment, but previous work has shown that this does not significantly improve accuracy and also increases computational cost.^25^ The EX0 method for exact exchange is therefore used throughout this work.

Previous work has also shown that in many situations, a block orthogonalization procedure is required to prevent an EMFT calculation from converging to a solution with unphysically low energy.^25,30^ This procedure involves forcing the off-diagonal blocks of the overlap matrix, i.e., *S*^AB^ and *S*^BA^, to be zero, by applying a transformation to the environmental basis functions to ensure they are orthogonal to the active region’s basis functions. For more details on block orthogonalization, see refs (25) and (30). This block orthogonalization procedure is applied throughout this work, and its effect on accuracy is discussed in section 3.1.

### 2.2 Time-Dependent Embedded Mean-Field Theory

Because EMFT is a mean-field theory, like DFT, TD-EMFT can be derived using a very similar process to that of standard linear-response TDDFT,^26^ which is briefly outlined in section S2 of the Supporting Information. The key quantities here are the exchange-correlation kernel *f*_xc_(**r**, **r**′) and the coupling matrix *Q*_cv,c′v′_,^33^ defined as
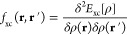
4
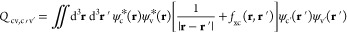
5*E*_xc_ is the exchange-correlation energy, ρ(**r**) is the electronic density, and ψ_v_(**r**) and ψ_c_(**r**) represent valence and conduction Kohn–Sham states, respectively. The Tamm–Dancoff approximation (TDA)^36^ (see section S2 of the Supporting Information for more details) is also used throughout this work, which makes calculations substantially more computationally efficient. Using the TDA can result in some errors in oscillator strengths relative to solving the full TDDFT problem but typically produces reliable excitation frequencies,^37^ which are the main properties of interest in this work.

In order to modify the standard TDDFT procedure for EMFT with DFT-in-DFT embedding, eq 3 implies that only changes to *f*_xc_ need to be considered, as the only thing that changes between the different levels of theory is *E*_xc_. Within EMFT, *E*_xc_^EMFT^[ρ] = *E*_xc_^low^[ρ] + (*E*_xc_^high^[ρ_AA_] – *E*_xc_^low^[ρ_AA_]). If this is substituted into eq 4, the result is
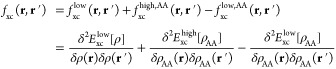
6

If this is followed through to the expression for the coupling matrix *Q* in eq 5, *Q* now becomes^26^
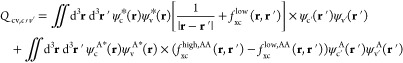
7where ψ^A^ represents the projection of a Kohn–Sham eigenstate onto the basis functions in region A alone. The TDDFT calculation can now proceed as usual but using the EMFT result for *Q* (eq 7) instead of the standard result (eq 5).

Similarly to ground-state DFT, to perform a TDDFT calculation with a hybrid functional, a fraction of exact exchange must be added to the coupling matrix *Q*. This contribution, *Q*^EX^, can be written as (in bra-ket notation)^23,33,38^
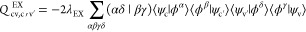
8

As above, ϕ are the basis functions, and ψ_v_ and ψ_c_ are the valence and conduction Kohn–Sham states, respectively. (αδ|βγ) is an electron repulsion integral (see ref (38)). λ_EX_ corresponds to the fraction of exact exchange included by the hybrid functional used. In a TD-EMFT calculation with a hybrid functional as the higher level of theory, this contribution must also be included but restricted, as before, to only include exchange within the active region:
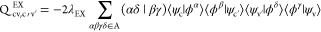
9

By replacing the contribution given by eq 8 with that in eq 9, established methods to solve the hybrid TDDFT problem can be used for TD-EMFT calculations.

### 2.3 TD-EMFT in ONETEP

In common with many other DFT codes, ONETEP uses a set of atom-centered basis functions to describe the system.^31^ What makes ONETEP different, however, is that these basis functions are not fixed; they are individually optimized to reflect the local environment of the atom on which they are situated, by optimizing the energy with respect to both the density kernel and the form of the basis functions themselves.^31^ Doing this allows for the basis set to be minimal in size, while still maintaining excellent accuracy. These basis functions, known as nonorthogonal generalized Wannier functions (NGWFs), are not required to be orthogonal to each other and are strictly localized, meaning they are defined to be zero beyond a certain radius from the atom they are centered on. This localization means that matrices, such as the Hamiltonian, are sparse, and therefore sparse matrix algebra can be used to improve the efficiency of the calculation. To allow for optimization, the NGWFs are defined on an underlying basis of psinc functions. The number of functions in this underlying basis is controlled by a cutoff energy, in an analogous way to the same quantity in plane-wave basis sets.

The details of the implementation of ground-state EMFT in ONETEP are presented in detail in ref (30), but here, one particular point of importance should be restated. As described in ref (30), although it is possible to optimize the NGWFs within an EMFT framework, the introduction of block orthogonalization (see section 2.1), significantly affects this optimization. Block orthogonalization effectively adds a new term to the gradient used to optimize the NGWFs; this new term competes with the other terms, leading to the optimization stalling. To avoid this, the NGWFs for the whole system are optimized at the lower level of theory (without imposing block orthogonalization) before fixing the NGWFs, block orthogonalizing them and optimizing the density kernel with EMFT. Although this means that the NGWFs are not completely optimized at the EMFT level, this gives an error in the total energy of less than 1%, which still provides excellent accuracy.^30^ The relative cost of this final optimization of the density kernel using EMFT varies depending on the system size and parallelization, but for the explicitly solvated phenolphthalein system discussed in section 3.2 and treated with PBE0-in-PBE EMFT, this step takes roughly twice as long as an optimization at the lower level of theory.

In a ONETEP ground-state energy calculation, the NGWFs are optimized to describe the occupied or valence Kohn–Sham states. This means there is no guarantee that these NGWFs will describe the unoccupied, or conduction, states, and indeed this is often the case.^31,39,40^ However, describing the conduction states well, or at least a subset of them, is vital for performing accurate calculations of excited-state properties. To remedy this, when such calculations are required, a new set of NGWFs is created to describe the conduction states. These conduction NGWFs are optimized to describe a given number of the lowest-lying conduction states by projecting the valence states out of the Hamiltonian. The original set of “valence” NGWFs and the new conduction NGWFs are then combined into a joint NGWF basis set that can describe both valence and conduction states.^40,41^ The same procedure is followed in a TD-EMFT calculation, simply projecting the valence states out of the EMFT Hamiltonian. As with the valence NGWFs, block orthogonalization is applied, implying that the conduction NGWFs are optimized at the lower level of theory only before fixing them and optimizing the conduction density kernel with EMFT.

Once a set of basis functions that can be used to correctly describe both valence and conduction Kohn–Sham states has been obtained, TDDFT calculations can be performed. TDDFT calculations in ONETEP follow the algorithm laid out in ref (33), which is briefly outlined in section S3 of the Supporting Information. To modify this algorithm for TD-EMFT, as in section 2.2, the usual expression for *f*_xc_ is replaced with the EMFT expression for this quantity, shown in eq 6.

Although it is not used in the results presented in this work, a feature of the TDDFT implementation in ONETEP relevant to TD-EMFT should still be emphasized. Because of the different levels of theory used to treat the active region and the environment in TD-EMFT, spurious excitations involving charge transfer between the two regions can become possible, particularly if the introduction of EMFT results in energy levels associated with different regions to swap their ordering. However, by truncating the response density kernel appropriately within ONETEP, it is possible to exclude particular types of excitations from the calculation, for example, nonphysical low-energy charge-transfer states that are a known issue with semilocal TDDFT.^4^ In particular, the excitations can be forced to be localized on a specific set of atoms by setting to zero any element of the response density kernel that involves a basis function not associated with these atoms. This would allow for spurious charge transfer between the regions in TD-EMFT, if present, to be eliminated, by localizing the excitations on the active region alone. However, the systems tested in this work do not exhibit such unphysical charge transfer excitations, and therefore no response kernel truncation is applied.

### 2.4 Combining (TD)-EMFT and Implicit Solvation

An implicit solvation model is included within ONETEP, using a minimal parameter solvent model based on the model of Fattebert and Gygi^42,43^ and extended by Scherlis et al.^35,44,45^ This model allows the solvent environment of the system under study to be described classically, as a polarizable dielectric medium. A cavity is defined around the system; at the edge of this cavity, there is a smooth transition in the value of the dielectric permittivity from the vacuum to the appropriate value for the solvent in question.^42^ The size and shape of the cavity is determined by the electronic density, typically a preliminary ground-state calculation is performed with the system in vacuum before defining and fixing the cavity at the size and shape implied by the density calculated in vacuum.^35^ The polarization induced in the solvent medium by the distribution of charge in the system can be calculated, and this in turn induces a new potential that is included in the Hamiltonian when optimizing the density kernel and NGWFs. This means that the electrostatic interaction between the solvent and the system can be included self-consistently when determining the ground-state energy, valence NGWFs, and conduction NGWFs as well as the excitation spectrum.

Using the implicit solvation model in ONETEP currently requires the use of open boundary conditions (OBCs), rather than the periodic boundary conditions (PBCs) typical in the rest of the code. Under these conditions, ONETEP uses the DL_MG library to calculate the total electrostatic potential by solving the generalized Poisson equation.^46^ DL_MG is a multigrid method. This solver also allows for the treatment of OBC calculations in vacuum within ONETEP, as such calculations just correspond to implicit solvent calculations with a solvent with a permittivity of 1.

For a given electronic density and cavity, the potential induced by the polarization of the solvent is independent of the functional used in the rest of the calculation. This means that the use of (TD-)EMFT does not affect the implicit solvation model directly, only indirectly by producing a different electronic density to the regular Hamiltonian. The main subtlety lies in how the cavity is defined. The preliminary calculation in vacuum used to determine this can be performed either at the lower level of theory or using EMFT. Both methods will give the same set of NGWFs (as NGWFs are optimized at the lower level of theory, as previously mentioned) but different density kernels. These two kernels will give slightly different cavities and potentially therefore different results. This difference is explored in sections 3.1 and 3.2.

## 3 Results

To test the implementation of TD-EMFT within linear-scaling DFT (specifically the code ONETEP), I have applied it to several different systems. In this section, each of these systems is described in turn, and the results of TD-EMFT calculations are reported, in order to validate and demonstrate the capabilities of the implementation. The .cif files for all structures shown are provided in the Supporting Information, converted using C2X.^47^ All spectra are broadened using lifetime broadening; ONETEP calculates the lifetime of each excitation and applies a Lorentzian broadening function to each excitation energy with a width corresponding to the appropriate lifetime.

### 3.1 Water–Nitrogen Dimer

**Figure 1:**
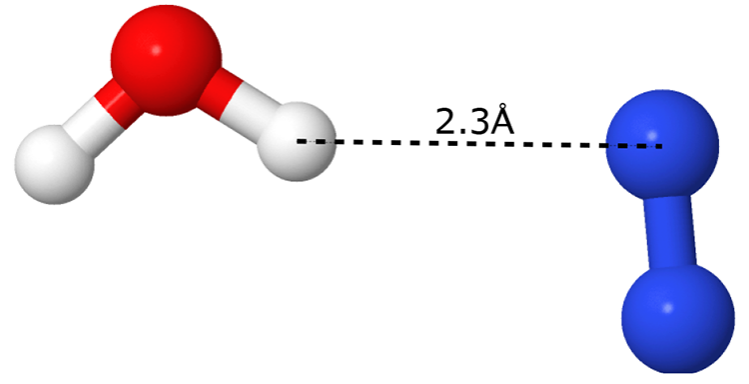
Structure of the water–nitrogen dimer used in this work. O, H, and N atoms are red, white, and blue, respectively. The shortest distance between the molecules is 2.3 Å, as labeled on the figure. Figure produced using Jmol.^48^

I first tested TD-EMFT as implemented within linear-scaling DFT on a very small and simple system, a dimer composed of a water molecule and a nitrogen molecule, separated by a distance of 2.3 Å. This structure is shown in Figure 1. I chose a dimer containing two different molecules rather than a water dimer to enable examination of any change in behavior when I changed which molecule is designated as the active region.

I examined the dimer both in vacuum and in implicit solvent, where the parameters of the implicit solvent are those appropriate for water near room temperature (permittivity ϵ_r_ = 78.54, surface tension γ = 0.074 15 N m^–1^). This allowed testing of the TD-EMFT implementation both with and without implicit solvent. I also looked at the effect of defining the implicit solvent cavity using the kernel optimized with EMFT (referred to as the EMFT cavity) or using the kernel optimized at the lower level of theory only (the non-EMFT cavity), as discussed in section 2.4.

The lower level of theory was chosen to be the local density approximation (LDA),^49,50^ while the higher level of theory was chosen to be the widely used hybrid functional B3LYP.^51^ Norm-conserving pseudopotentials distributed with ONETEP were used for all three species. A cutoff energy of 850 eV was used, and NGWF radii of 11 bohr were used for all species. Four NGWFs were associated with each of the O and N atoms and one with the H atoms. The dimer was centered in a large cubic cell, with side lengths of 75 bohr. The vacuum calculations were performed under PBCs in this cell, while the implicit solvent calculations were performed under OBCs. In the vacuum calculations, the large size of the cell eliminates interaction between the dimer and its periodic images, meaning that they are directly comparable to the implicit solvent calculations. OBC calculations within ONETEP must make use of the DL_MG multigrid solver, which reduces their computational efficiency somewhat, so large-cell PBC calculations are preferred where possible.

Most of the results presented here are focused on the lowest energy reasonably bright excitation of the water–nitrogen dimer, which ranges between 5 and 7 eV in vacuum depending on the method used. This excitation is not the strongest in the spectrum of this system; there is another brighter excitation that ranges between 6.5 and 7.5 eV in vacuum. However, focusing on the lower energy excitation allows for a more thorough test of TD-EMFT, as the difference between LDA and B3LYP is very pronounced for this excitation. This difference is not as large for the higher energy excitation, although the same conclusions can be drawn from both. The higher energy excitation is discussed at the end of the present section, as well as in section S4 in the Supporting Information.

The effect of the block orthogonalization (BO) protocol discussed in section 2.1 can be identified by comparing a standard LDA TDDFT calculation and an LDA-in-LDA TD-EMFT calculation. An LDA-in-LDA TD-EMFT calculation treats all parts of the system at the same level of theory (LDA) but does so using the machinery of EMFT, including, importantly, BO; this means that any difference between the calculations can be ascribed to the presence of BO. A comparison between these calculations for the water–nitrogen dimer shows there is excellent agreement. The difference in ground state energies is 1.3 and 2.4 meV in vacuum and solvent respectively, well within acceptable limits. This is also the case for the low-energy excitation; the difference in excitation energy is 2.1 and 3.3 meV in vacuum and solvent, respectively. This demonstrates that BO does not significantly affect the accuracy of the results. The conduction NGWFs seem to be more sensitive to the presence of BO, and if the “ground state” energy of the projected Hamiltonian used to optimize the conduction NGWFs^40^ is compared, the difference is 52 and 48 meV in vacuum and solvent, respectively. However, this level of agreement is still more than sufficient to obtain matching solutions to the TDDFT problem, as already seen.

**Figure 2:**
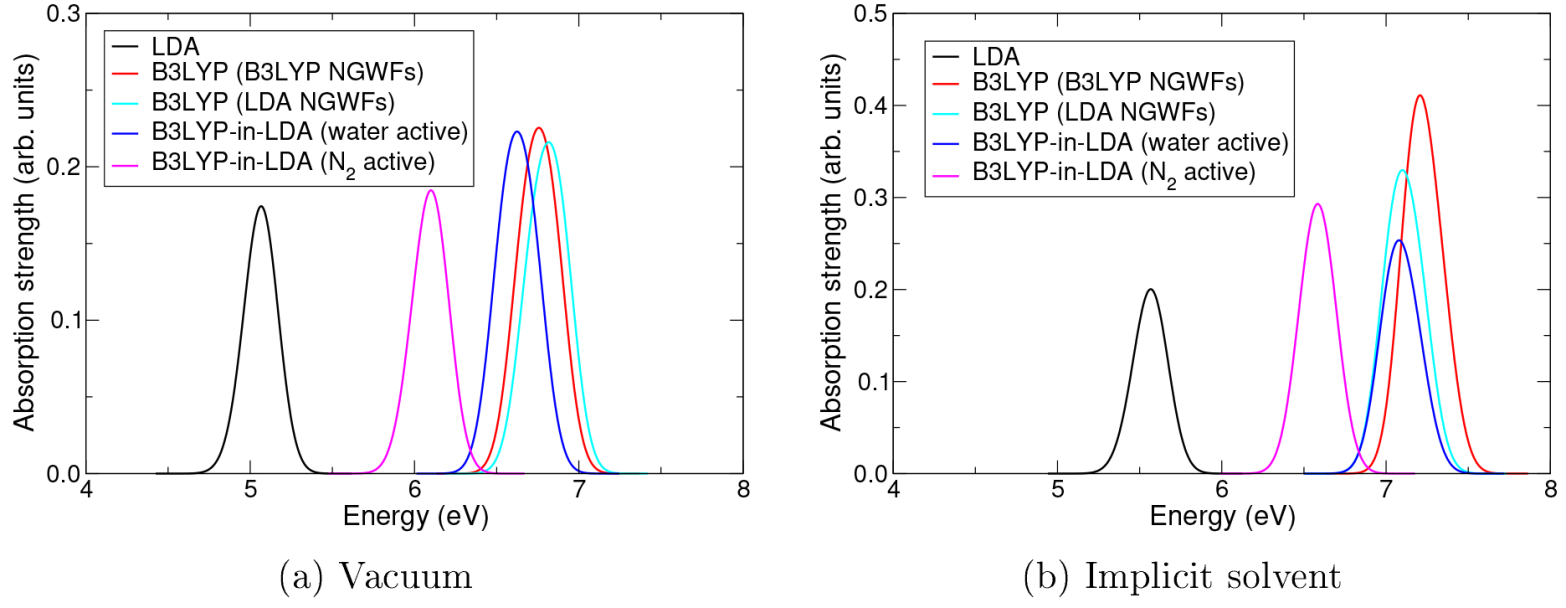
Low-energy absorption spectra of the water–nitrogen dimer calculated at various levels of theory: full system LDA (black), full system B3LYP with LDA-optimized NGWFs (cyan), full system B3LYP with B3LYP-optimized NGWFs (red), B3LYP-in-LDA with water as the active region (blue), and B3LYP-in-LDA with nitrogen as the active region (magenta). (a) Results calculated in vacuum and (b) results calculated in implicit solvent (water), using the non-EMFT cavity.

Figure 2 presents the calculated low-energy absorption spectra; Figure 2a shows the results in vacuum, while Figure 2b shows them in implicit solvent using the non-EMFT cavity. The low-energy absorption spectrum is calculated using LDA for the whole system (black line in figures), B3LYP for the whole system (red/cyan), and B3LYP-in-LDA EMFT, with either the water or nitrogen molecule acting as the active region (blue and magenta, respectively). When the whole system is treated with B3LYP, two sets of results are presented, one using NGWFs optimized at the B3LYP level (red), as in a normal ONETEP calculation, and one using NGWFs optimized at the LDA level, as in an EMFT ONETEP calculation (cyan).

As expected, LDA produces a significantly lower excitation energy than B3LYP in all cases; this is precisely the discrepancy TD-EMFT aims to correct. It can also be seen that the B3LYP calculations performed with LDA- and B3LYP-optimized NGWFs agree well; the excitation energy calculated with LDA-optimized NGWFs is within 0.05 eV of that obtained in the pure B3LYP calculation in vacuum and within 0.13 eV in implicit solvent. This demonstrates that the error introduced by using NGWFs optimized at the lower level of theory does not significantly affect the accuracy of the calculation, especially when compared to the difference between the lower and higher levels of theory, validating this approximation within the TD-EMFT calculations.

However, the most important feature of the spectra shown in Figure 2 is that the B3LYP-in-LDA results agree well with the full system B3LYP results, if the water molecule is taken as the active region. If the water molecule is treated with B3LYP, TD-EMFT calculations give an error compared to the full B3LYP results of 0.13 eV in vacuum and 0.15 eV in implicit solvent, comparable to the error arising from using LDA-optimized NGWFs. If instead the nitrogen molecule is taken as the active region, this error becomes significantly worse, although the resulting excitation energy is still significantly closer to the B3LYP value than the LDA value. The reasons for this are discussed in more detail below, with reference to Figure 4. The oscillator strength of the excitation also varies a little as the level of theory is changed, although this is a secondary concern as the oscillator strength is less reliably calculated under the TDA anyway. Taken together, these results validate the accuracy of the TD-EMFT method, and in particular the implementation of it in ONETEP, as long as the active region is chosen wisely.

**Figure 3:**
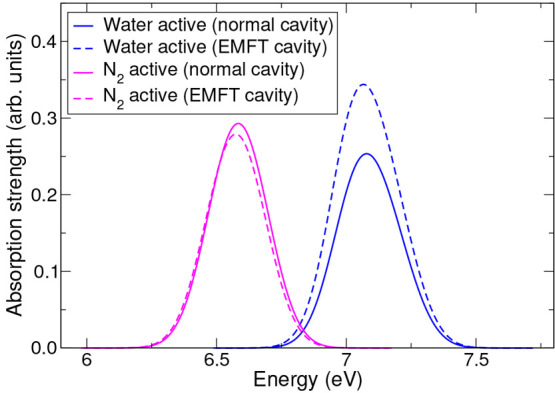
Low-energy absorption spectra of the water–nitrogen dimer calculated using B3LYP-in-LDA TD-EMFT in implicit solvent, using either the non-EMFT (solid lines) or EMFT cavity (dashed lines). The results for both B3LYP-in-LDA with water as the active region (blue lines) and B3LYP-in-LDA with nitrogen as the active region (magenta lines) are presented.

Examining the effect of implicit solvent on our calculations, comparing parts a and b of Figure 2 shows that the introduction of implicit solvent induces a blue shift of roughly 0.5 eV at every level of theory and also has some effect on the oscillator strengths. The overall accuracy of the TD-EMFT method, however, is not significantly affected by the presence of implicit solvent, demonstrating that TD-EMFT and implicit solvent can be used successfully together. Figure 3 shows that changing whether the cavity is created using the LDA- or EMFT-optimized density kernel makes very little difference to the excitation energies but can change the absorption strengths. This is likely a symptom of the fact that the active region is at the edge of the cavity, so the change in kernel will directly affect the shape and size of the cavity. This is not a particularly likely mode of operation; in more realistic systems, such as the phenolphthalein system in section 3.2, the active region will be surrounded by the lower-level environment region and will therefore not be close to the edge of the cavity. In such systems, results obtained with the EMFT and non-EMFT cavities would be expected to be extremely similar. It is reassuring, however, that even in the case where the active region does lie at the edge of the cavity, the choice of cavity does not affect the accuracy of the most important property, the calculated excitation energies.

**Figure 4:**
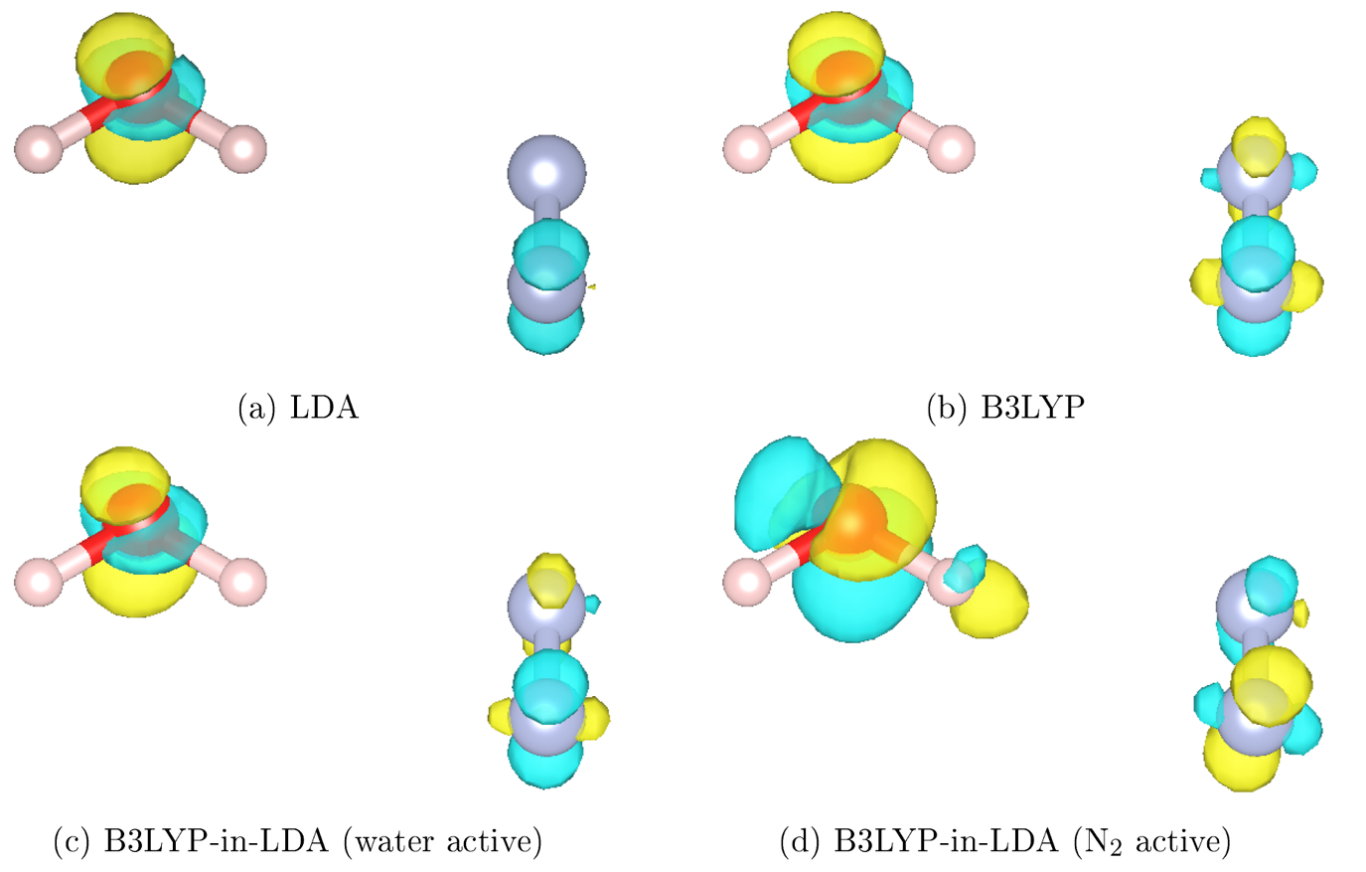
Isosurfaces of the calculated response density for the excitation of the water–nitrogen dimer seen in Figure 2b, calculated at various levels of theory: full system LDA, full system B3LYP (with B3LYP-optimized NGWFs), B3LYP-in-LDA with water as the active region, and B3LYP-in-LDA with nitrogen as the active region. All response densities were computed with implicit solvent, using the non-EMFT cavity. The isosurfaces are at |*n*| = 0.01 e Å^–3^, with yellow and blue representing positive and negative response densities, respectively. O, N, and H atoms are red, blue, and white, respectively. Figures produced using VESTA.^52^

The results shown in Figures 2 and 3 can be more clearly understood by looking more closely at the character of these excitations. Figure 4 shows isosurfaces of the response density for the excitation calculated at different levels of theory. It is immediately obvious that in all cases, the excitation has character on both the water and nitrogen molecules but that the response density is higher near the water molecule. This implies that the excitation is more associated with the water molecule than the nitrogen molecule. This fits with Figure 2, where the B3LYP-in-LDA results are much closer to the full B3LYP results when the water molecule is the active region. The excitation looks very similar in the LDA, B3LYP, and B3LYP-in-LDA (active water) calculations (Figure 4a, b, and c, respectively). This emphasizes that if the most important region is treated at the higher level of theory, an accurate description of the system can be obtained with TD-EMFT. However, if the nitrogen molecule is treated as the active region instead, the excitation changes quite significantly, as can be seen in Figure 4d. This shows that describing the wrong part of the system at the higher level of theory can change the nature of the excitation. Because the excitation does have some character on the nitrogen molecule, the active nitrogen TD-EMFT calculation would be expected to correct the LDA excitation energy to some extent, as can be seen in Figure 2a, but not to the same extent as the active water TD-EMFT calculation. This also implies that some of the error in the B3LYP-in-LDA TD-EMFT results likely comes from the incorrect treatment of the part of the excitation that is localized on the molecule treated at the lower level of theory; this is more of a problem when the nitrogen molecule is the active region, as previously discussed.

The discussion above focuses on the low-energy excitations of the water–nitrogen dimer, as noted previously, but it also applies to the higher energy bright state found between 6.5 and 7.5 eV in vacuum. Figures S1 and S2 in the Supporting Information give results for this higher energy excitation, comparable to Figures 2a and 4, respectively. In this case, the difference between the energies predicted by LDA and B3LYP (with B3LYP-optimized NGWFs) is 0.45 eV, significantly smaller than before. This means that there is not as much to gain from utilizing TD-EMFT, as LDA describes this excitation much better than the lower energy excitation treated previously. However, even with this caveat, B3LYP-in-LDA TD-EMFT is significantly closer to the full B3LYP result, demonstrating the power of TD-EMFT. When water is the active region, the error is 0.10 eV, which is actually less than the error from using LDA-optimized NGWFs in a B3LYP calculation (0.15 eV). Unlike before, the error in B3LYP-in-LDA calculations with nitrogen as the active region (− 0.09 eV) is comparable to calculations with water as the active region. This is likely partially due to this higher energy excitation having more response density on the nitrogen molecule, as seen in Figure S2 in the Supporting Information. Although the gains are smaller, these data demonstrate the utility of TD-EMFT for the higher excitation as well. For more discussion on this, see section S4 in the Supporting Information.

### 3.2 Phenolphthalein in Water

**Figure 5:**
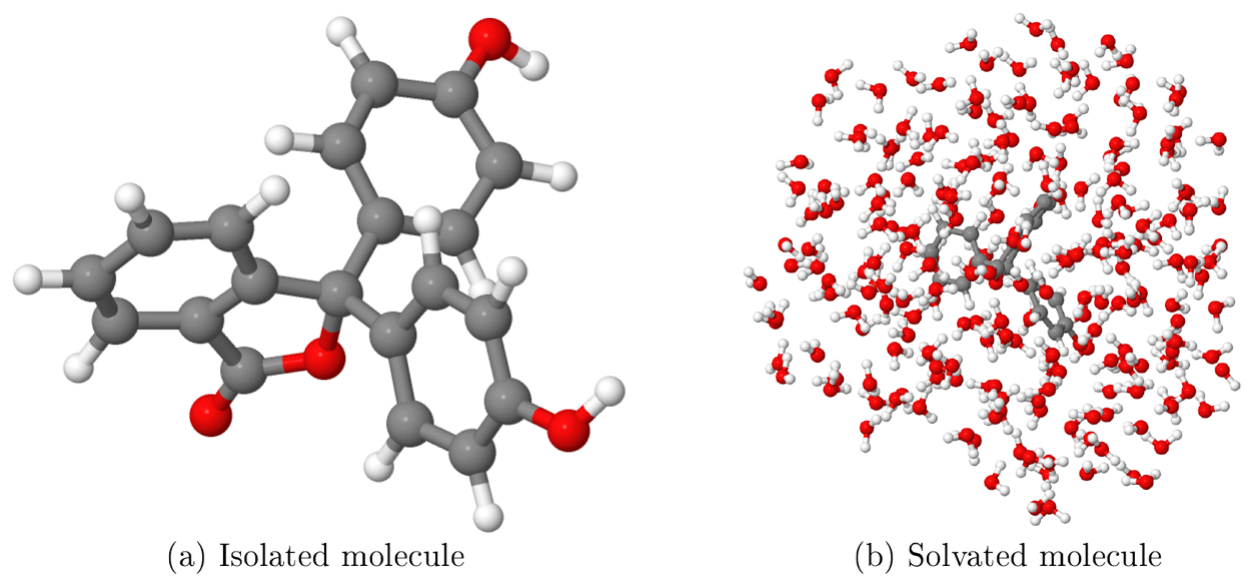
Structures used for the calculations on phenolphthalein in this work: (a) isolated phenolphthalein molecule, in its neutral charge state and (b) same molecule explicitly solvated with 218 water molecules, as extracted from a classical molecular dynamics simulation. C, O, and H atoms are gray, red, and white, respectively. Figures produced using Jmol.^48^

I next applied linear-scaling TD-EMFT to the case of the molecule phenolphthalein solvated in water. Phenolphthalein is a well-known pH indicator. For pH values up to around 8–9, the molecule is in a charge-neutral configuration that is colorless, but at higher pH, it donates two protons, becoming doubly negatively charged. This changes the chemical structure of the molecule and leads to it exhibiting a fuschia-pink color.^53^ In this work, I focused on the neutral configuration, which is shown in Figure 5a.

Phenolphthalein is typically used as an indicator in solution, usually water. In order to accurately describe the absorption spectrum of phenolphthalein, therefore, the effect of the solvent must be accurately described. This requires not only an excellent quantum mechanical description of the interaction between the solvent and solute but also an accurate description of the configuration of the water molecules around the solvent. TD-EMFT can help with the former requirement, but the latter typically requires molecular dynamics (MD) calculations. Previous work has shown that the effect of the solvent on absorption spectra can be sensitive to very long-range interactions, meaning such large system sizes are required as to make *ab initio* MD impractical.^4^ Instead, appropriate configurations are best obtained by conducting *classical* MD simulations of the solvated system, taking snapshots from the resulting trajectory, carving out a section around the solute to treat with TDDFT, and averaging the results over all snapshots.^4,54^ This procedure was followed in this work, although as the aim was not to converge my results with respect to the number of snapshots, I considered only a single snapshot. The structure of this snapshot is shown in Figure 5b. The precise procedure used to obtain this structure is detailed in section S5 of the Supporting Information.

I computed the absorption spectrum of phenolphthalein in water using several different methods. First, the isolated phenolphthalein molecule in implicit solvent was treated with both the semilocal functional PBE^55^ and the hybrid functional PBE0,^56^ with no embedding involved. In this case, the PBE0 hybrid functional was used rather than B3LYP, as previous unpublished calculations on this system using the spectral warping approach^54^ rather than TD-EMFT (and including significant levels of sampling) suggested that PBE0 performs better in comparison to experiment. For the PBE0 calculation, PBE-optimized NGWFs were used for consistency with the other calculations.

**Figure 6:**
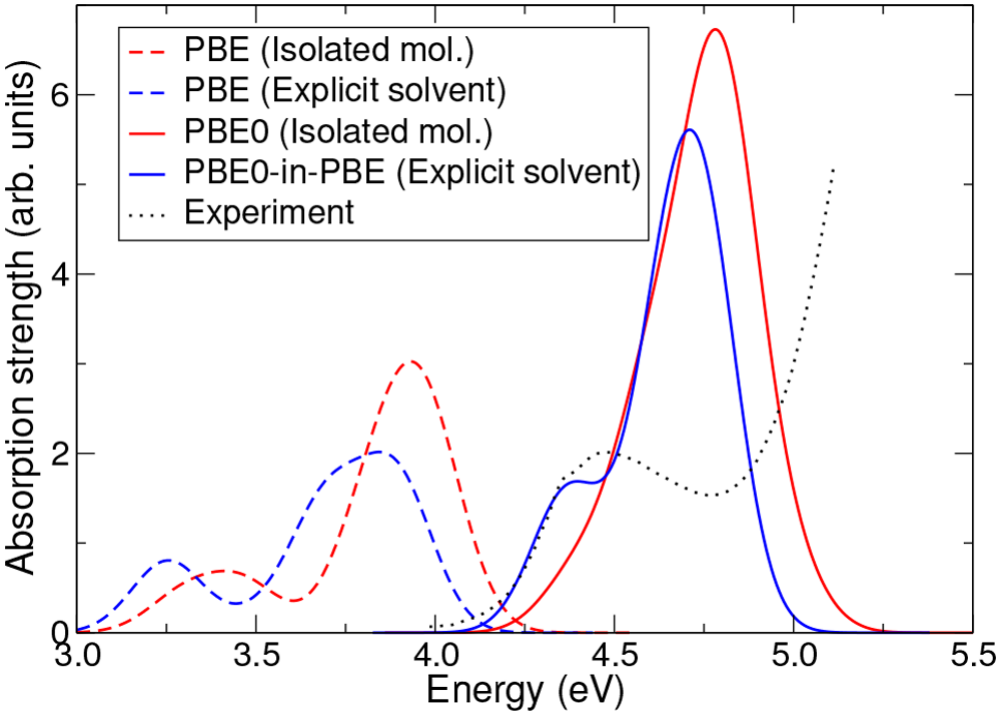
Absorption spectra of neutral phenolphthalein in water as calculated using various different methods. The red curves correspond to the results obtained for the isolated molecule in implicit solvent only (see Figure 5a), while the blue curves correspond to the results obtained with the explicitly solvated system (see Figure 5b). Dashed lines correspond to the results obtained using the PBE functional, while solid lines correspond to the results obtained using the PBE0 functional (with PBE-optimized NGWFs) for the isolated molecule and PBE0-in-PBE TD-EMFT for the explicitly solvated system, with phenolphthalein as the active region. The black dotted line shows the experimental absorption spectrum from ref (58). All calculations are performed in implicit solvent, using the non-EMFT cavity.

I then considered the explicitly solvated phenolphthalein system; all calculations containing explicit solvent were also placed in implicit solvent, giving both an explicit and an implicit layer of solvent. I calculated the absorption spectrum of the solvated system with pure PBE, and then with TD-EMFT, using PBE and PBE0 as the lower and higher levels of theory, respectively, with the phenolphthalein molecule as the active region. In addition to these calculations (the results of which are presented in Figure 6), I performed several others to investigate the interplay between the amount of explicit solvent included and the level of theory used to describe it. Performing a full PBE0 calculation on the entire explicitly solvated system would be extremely computationally demanding and therefore is not attempted here.

Norm-conserving pseudopotentials produced using the atomic solver of the plane-wave pseudopotential DFT code CASTEP^57^ were used for all species; details of these pseudopotentials can be found in section S1 of the Supporting Information. A cutoff energy of 800 eV was used throughout. The NGWF radii were different for the solvent molecules and the solute itself. In the solvent molecules, H and O had valence NGWF radii of 7 and 9 bohr, respectively, and conduction NGWF radii of 7 and 11 bohr, respectively. In the solute, all NGWFs had a radius of 11 bohr. 4, 4, and 1 NGWFs were associated with C, O, and H atoms in all parts of the system. As noted above, all the calculations were performed in implicit solvent, with the parameters appropriate for water. All calculations were performed in a cubic cell with a side length of 75 bohr.

**Figure 7:**
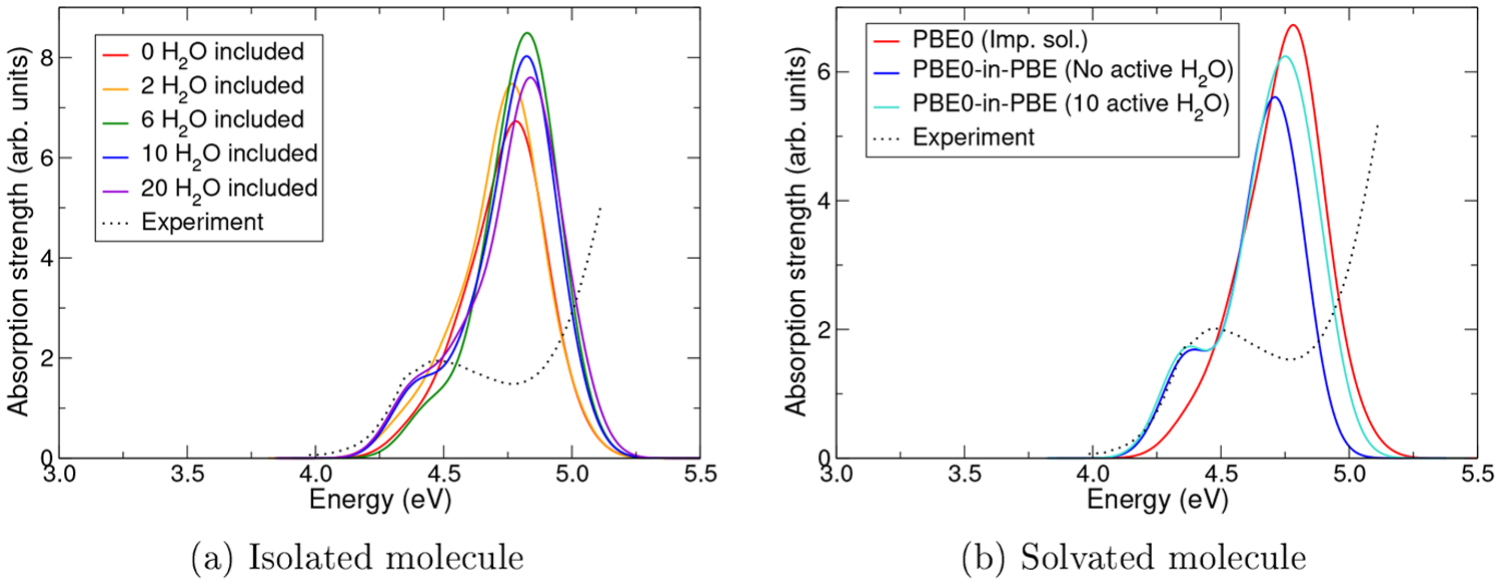
Absorption spectra of neutral phenolphthalein in water as calculated using various different methods: (a) absorption spectra calculated using the PBE0 functional (with PBE-optimized NGWFs) with increasing numbers of nearby water molecules explicitly included. The colors of the curves progress through the rainbow (red → orange → green → blue → violet) as the number of explicit water molecules increases. (b) Absorption spectra calculated using the PBE0 functional for isolated phenolphthalein; PBE0-in-PBE TD-EMFT for explicitly solvated phenolphthalein, with the phenolphthalein as the active region; and PBE0-in-PBE TD-EMFT for explicitly solvated phenolphthalein, with the phenolphthalein molecule and the 10 nearest water molecules as the active region. The first two of these spectra are also presented in Figure 6. In both parts a and b, the black dotted line shows the experimental absorption spectrum from ref (58). All calculations are performed in implicit solvent, using the non-EMFT cavity.

Figures 6 and 7 show the absorption spectra calculated by the various methods detailed above as well as experimental data^58^ for comparison. The experimental data exhibits two clearly separated peaks, with the higher energy peak significantly larger than the lower. Along with the excitation energy of these peaks, this two-peak structure is something that calculations should replicate in order to describe the system accurately.

The structure of the spectrum obtained with PBE (dashed lines in Figure 6) exhibits a two-peak structure, both for the isolated molecule and the explicitly solvated system, although the excitation energies of the peaks are much lower than in experiment, as expected. This provides reassurance that the lower level of theory (PBE) is describing the system qualitatively correctly. The explicitly solvated system is red-shifted by around 0.11 eV compared to the isolated molecule, and the lower energy peak is relatively stronger compared to the higher energy peak. Looking at the PBE0/PBE0-in-PBE spectra (solid lines in Figure 6), however, it can be seen that the two-peak structure remains for the explicitly solvated PBE0-in-PBE calculation but disappears for the isolated molecule PBE0 calculation (in fact; the lower energy peak remains but is much smaller and is subsumed by the larger higher energy peak). The excitation energies are now much closer to the experimental results than in the PBE case; in particular, the PBE0-in-PBE value for the excitation energy of the lower peak is within 0.11 eV of that seen in the experimental data, although the energy of the higher peak is significantly below that seen in the experimental data. The red-shift due to explicit solvation is of a similar magnitude to the PBE case (0.14 eV for the higher energy peak).

The quantitative accuracy of the excitation energies could potentially be improved by using an optimally tuned range-separated hybrid functional rather than PBE0,^59^ but such functionals are not yet available in ONETEP, so they are not considered here. It should also be noted that exact agreement with experiment is not to be expected, as I have only looked at a single snapshot, rather than averaging over many; however, using TD-EMFT to calculate the absorption spectrum gives reasonable excitation energies, while also maintaining the clear two-peak structure seen in experiment. Giving a qualitatively correct description of the physics of the system alongside reasonable quantitative predictions is not something that is achieved by any of the other methods examined here.

To further examine the effect of including water molecules explicitly in our calculations, and therefore demonstrating the utility of TD-EMFT over implicit solvent or similar calculations, further calculations treating water molecules with various levels of theory were performed. First, starting from the PBE0 isolated phenolphthalein calculation already presented in Figure 6, I considered including explicit water molecules in this calculation (also treated with PBE0), with the molecules introduced in order of proximity to the phenolphthalein molecule. Figure 7a shows the absorption spectra obtained including 0, 2, 6, 10, and 20 water molecules in this way. The number of water molecules was limited to a maximum of 20 due to the computational expense of treating more molecules with PBE0. Second, I performed a PBE0-in-PBE TD-EMFT calculation on the explicitly solvated system, similar to that already presented in Figure 6, but this time with the 10 water molecules closest to the phenolphthalein included in the active region. The result of this calculation is presented in Figure 7b.

The results of Figure 7a show that the two-peak structure becomes more distinct as more explicit water is included; this can be seen most extremely by comparing the isolated molecule and explicitly solvated systems in Figure 6, as previously noted. The explicit inclusion of the water has a qualitative effect on the spectrum, as seen in previous work.^4^ The results of Figure 7b then imply that the influence of the explicit water molecules is relatively unaffected by the level of theory used to describe them, as there is very little difference between the spectrum resulting from treating nearest neighbor water molecules with PBE0 and the spectrum where only the phenolphthalein is treated with PBE0. Taking the subfigures together, Figure 7 forms a strong argument for the utility of TD-EMFT in this system: including a large number of water molecules is necessary to correctly qualitatively describe the system, but the results are relatively insensitive to the level of (quantum mechanical) theory used to do this, so a lower level of theory can be used. It is important that the environment is treated quantum mechanically, rather than classically, a point that is backed up by previous comparisons to QM/MM methods.^4^ Overall, the results of the calculations presented in this section demonstrate that the implementation of TD-EMFT with implicit solvent within linear-scaling DFT is able to successfully describe a complex system containing several hundred atoms, giving a qualitatively correct description of the system and reasonably accurate quantitative results.

I also investigated the effect of using the EMFT cavity rather than the non-EMFT cavity in the PBE0-in-PBE calculation but found this made effectively no difference to the results, changing the peak excitation energies by less than 1 meV and the oscillator strengths by less than 0.6%. As outlined in section 3.1, this is as expected, as the active region is now not close to the edge of the cavity, so any change in the density kernel due to EMFT is likely to be localized far from the cavity edge.

The results of this section also demonstrate an important point regarding the savings TD-EMFT provides. The main limiting factor for hybrid calculations in ONETEP is computer memory, rather than speed. This means that the savings in memory that (TD-)EMFT provides are as important, if not more, than any speed-up. This is demonstrated by the fact that I was unable to reasonably perform a full hybrid TDDFT calculation on a system containing a phenolphthalein molecule and more than 20 explicit water molecules due to memory constraints, but I was able to perform a calculation containing significantly more water molecules using TD-EMFT.

### 3.3 Pentacene in *p*-Terphenyl

Finally, I applied linear-scaling TD-EMFT to the pentacene-doped *para*-terphenyl molecular crystal. The author and co-workers also studied this system in our previous work on ground state EMFT,^30^ allowing comparisons to be drawn easily. This system can be used as the basis of a room-temperature maser,^60^ – as most previously known masing systems only work under stringent operating conditions,^61−63^ and this system has many important potential applications. Although the population inversion necessary for masing behavior is actually formed between different spin states of the triplet ground state of the pentacene molecule (*T*_1_), these states are populated via a route that starts with exciting pentacene molecules into the first excited singlet state (*S*_1_) from the (singlet) ground state (*S*_0_).^60,64^ This means that the *S*_0_ to *S*_1_ transition energy () is very important and is the focus here.

**Figure 8:**
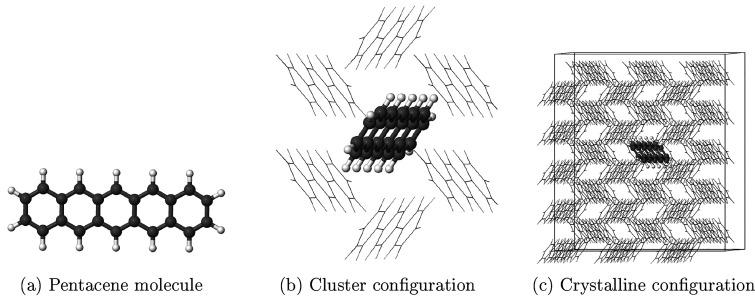
Structures used for the calculations on the pentacene in the *p*-terphenyl system in this work: (a) isolated pentacene molecule, (b) cluster configuration, which is made up of a pentacene molecule and its six nearest neighbor *p*-terphenyl molecules in a herringbone structure, and (c) crystalline configuration. This structure is constructed by taking a 3 × 5 × 3 supercell of crystalline *p*-terphenyl and replacing the central *p*-terphenyl with a pentacene molecule. This structure is periodic, with the unit cell also indicated in the figure. In parts b and c, the *p*-terphenyl molecules are shown as wireframes, and the pentacene is shown using a ball-and-stick model. H and C atoms are white and gray, respectively. Figures reproduced with permission from ref (30). Copyright 2020 American Chemical Society.

The presence of *p*-terphenyl has significant effects on the excitation energies of the pentacene molecule,^64−68^ and therefore it is important to include the *p*-terphenyl environment in our calculations. The question of how much of the environment to include is, however, a more difficult question. To this end, three different configurations are considered, as in previous work,^30^ an isolated pentacene molecule in vacuum, a cluster model containing the pentacene molecule and its 6 nearest *p*-terphenyl neighbors, and a crystalline model corresponding to a 3 × 5 × 3 supercell of *p*-terphenyl with the central molecule substituted with pentacene. These three structures are shown in Figure 8a–c. TD-EMFT is applied to the cluster and crystalline structures, taking the pentacene molecule as the active region and the surrounding *p*-terphenyl molecules as the environment. The crystalline structure in particular contains 2884 atoms, allowing linear-scaling TD-EMFT to be tested on a (previously unattainable) very large system, thus including long-range interactions with the environment.

In the solvated phenolphthalein system studied in section 3.2, it was clear that a quantum mechanical description of the environment out to long ranges, rather than a classical description, was important. We expect this to still apply to some degree in the crystalline system here, meaning that the full crystalline calculation should describe the system more accurately than a similar one using a QM/MM method. Although QM/MM methods have been applied to the pentacene-in-*p*-terphenyl system in recent work,^69^ the use of semiempirical density functional tight binding methods for the QM region makes it difficult to draw comparisons with the present work, as the strong dependence of the excitations on the QM method overwhelms any effect from the classical description of the environment.

In previous work on ground state EMFT,^30^ the author and co-workers were able to estimate  using a combination of the ΔSCF method to obtain Δ*E*_*S*_0_→*T*_1__ and the Becke method^70^ to obtain Δ*E*_*T*_1_→*S*_1__ for each of the three structures. The *S*_0_ to *S*_1_ transition has also been previously studied with TDDFT: an isolated molecule in vacuum, treated with PBE (Δ*E*_*S*_0_→*S*_1__ = 1.64 eV), B3LYP (1.90 eV),^71^ and an optimally tuned range-separated hybrid functional (OT-LCωPBE, 2.15 eV);^64^ an isolated molecule in *p*-terphenyl-like implicit solvent, treated with PBE (1.60 eV) and OT-LCωPBE (2.07 eV),^64^ or treated with PBE and empirically corrected (2.27 eV);^68^ and the cluster configuration, treated with PBE (1.58 eV) and OT-LCωPBE (2.09 eV).^64^ The results of this work can be compared to these previous results and also to experimental data.^65,72^

Here, PBE is used as the lower level of theory, with B3LYP as the higher level. The norm-conserving pseudopotentials distributed with ONETEP were used for both species, the cutoff energy was taken as 750 eV, and the NGWF radii were set to 11 bohr for all atoms, with 4 NGWFs associated with the C atoms and 1 with the H atoms. All three configurations were performed in PBCs, with the same unit cell (that of the 3 × 5 × 3 supercell of *p*-terphenyl).

Table 1 presents the results of the calculations on the three structures, alongside experimental data.^65,72^Figure S3 in section S6 of the Supporting Information shows the absorption spectra corresponding to the same data. It can immediately be seen that the B3LYP-in-PBE TD-EMFT calculations for both the cluster and crystal configurations match well with crystalline experimental data; in fact, the crystalline calculation matches the experiment almost exactly, with the cluster calculation 0.02 eV lower. This demonstrates the importance of including long-range interactions between the pentacene and its environment. The ordering of the three configurations in terms of excitation energy (cluster, crystal, vacuum) and in terms of absorption strength (crystal, vacuum, cluster) is the same for both PBE and B3LYP/B3LYP-in-PBE. These results provide evidence that linear-scaling TD-EMFT is correctly describing the excitation and gives quantitatively accurate results for systems containing thousands of atoms. The B3LYP-in-PBE TD-EMFT calculations also produce values for Δ*E*_*S*_0_→*S*_1__ significantly closer to experiment than the indirect method used in previous work (1.85 and 1.76 eV for the cluster and crystal configurations, respectively).^30^ This demonstrates the utility of using TD-EMFT directly, rather than only ground state EMFT.

**Table 1:** Excitation Energies Calculated Using TDDFT/TD-EMFT for the Transition between the *S*_0_ and *S*_1_ States for Pentacene, In the Three Geometries Shown in Figure 8^a^

	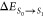 (eV)		
configuration	PBE	B3LYP-in-PBE	exptl
vacuum	1.880	2.198	2.31^72^
cluster	1.792	2.069	2.09^65^
crystal	1.810	2.089	

a Experimental data from refs (65 and 72) are also shown. For the cluster and crystal configurations, B3LYP-in-PBE refers to a TD-EMFT calculation, whereas for the vacuum configuration, it corresponds to a B3LYP calculation performed using PBE-optimized NGWFs.

It should also be noted that the vacuum calculations, including those done with B3LYP, underestimate the value measured experimentally in vacuum. This is in line with previous computations performed with hybrid DFT, even when using an optimally tuned range-separated hybrid functional.^64^ Higher-order methods such as multireference Møller–Plesset perturbation theory do give the correct excitation energy^73^ but are not implemented within the EMFT framework within ONETEP and are therefore not considered here. Hybrid functionals such as B3LYP provide a more reliable description of the excitation spectrum in the solid state, where screening reduces the HOMO–LUMO gap, bringing it in line with hybrid functional predictions.^74^

## 4 Conclusions

In this work, I have presented the first implementation of time-dependent embedded mean field theory combined with both linear-scaling density functional theory and a classical implicit solvation model, all within the linear-scaling DFT code ONETEP. This combination allows for multilevel simulations of electronic excitations of large-scale systems to be conducted, with two levels of DFT and a classical continuum model all contained within the same calculation. Such calculations will likely be extremely useful in systems where excitations of interest are largely localized on a particular active region, but the environment affects these excitations both quantum mechanically and classically. I have demonstrated the power and utility of this method by applying it to a wide range of different systems, including the water-nitrogen molecular dimer, phenolphthalein in water, and pentacene-doped *p*-terphenyl. In each case, the linear-scaling TD-EMFT method obtains excellent results, agreeing well with experimental data and previous calculations. These calculations also demonstrated that the method can be used for systems containing thousands of atoms, which would not have previously been accessible for purely high-accuracy hybrid functional TDDFT. This work will allow embedding calculations of electronic excitations to be applied to an even wider range of problems than previously, both in terms of scale and also in terms of systems of interest in physics, chemistry, and materials science.

## Supporting Information Available

Structural information for water–nitrogen dimer (CIF)
Structural information for isolated phenolphthalein molecule (CIF)
Structural information for phenolphthalein molecule explicitly solvated with water (CIF)
Structural information for isolated pentacene molecule (CIF)
Structural information for pentacene-doped *p*-terphenyl cluster (CIF)
Structural information for pentacene-doped *p*-terphenyl crystal (CIF)
Further details of pseudopotentials used in this work, outline of derivation of TDDFT equations and algorithm to solve them as used in this work, additional absorption spectra data for water–nitrogen dimer, further details on method for obtaining snapshot used for phenolphthalein in water calculations, and absorption spectra for pentacene in *p*-terphenyl (PDF)

## Supplementary Material

ct1c01133_si_001.cif

ct1c01133_si_002.cif

ct1c01133_si_003.cif

ct1c01133_si_004.cif

ct1c01133_si_005.cif

ct1c01133_si_006.cif
