# Supplementary material for: Efficiently Computing Excitations of Complex Systems: Linear-Scaling Time-Dependent Embedded Mean-Field Theory in Implicit Solvent
Source: J Chem Theory Comput. 2022 Feb 8;18(3):1542–54. doi: 10.1021/acs.jctc.1c01133 (PMC9082505; doi:10.1021/acs.jctc.1c01133)
Supplement: Supplementary file 7 — ct1c01133_si_007.pdf [file ct1c01133_si_007.pdf]

Supporting Information for Efficiently computing the excitations of complex systems: linear-scaling time-dependent embedded mean-field theory in implicit solvent

Joseph C. A. Prentice

January 13, 2022

S1 Psuedopotentials

The pseudopotential strings defining the pseudopotentials used in the main text for the calculations on phenolphthalein are:

- C: 1|1.2|17|20|23|20N:21L(qc=8)
- O: 1|1.2|23|26|31|20N:21L(qc=9)
- H: 1|0.8|14|16|19|10N(qc=8)

S2 Derivation of TDDFT equations

If the ground state of the system is perturbed by a small time-dependent perturbation with frequency ω, $\delta v(\mathbf{r},\omega)$, the change in the density $\rho(\mathbf{r})$ to first order (the linear response) will be

$$\delta\rho(\mathbf{r},\omega) = \int d^3\mathbf{r}'\,\chi(\mathbf{r},\mathbf{r}',\omega)\delta v(\mathbf{r}',\omega)\ . \tag{1}$$

Here, χ is the density-density linear-response function

$$\chi(\mathbf{r},\mathbf{r}',\omega) = \sum_{v,c}\left[\frac{\psi_v^*(\mathbf{r})\psi_c(\mathbf{r})\psi_v(\mathbf{r}')\psi_c^*(\mathbf{r}')}{\omega-(\epsilon_c-\epsilon_v)} - \frac{\psi_v(\mathbf{r})\psi_c^*(\mathbf{r})\psi_v^*(\mathbf{r}')\psi_c(\mathbf{r}')}{\omega+(\epsilon_c-\epsilon_v)}\right]\ , \tag{2}$$

where the ψ_v and ψ_c are the valence and conduction ground-state Kohn-Sham eigenstates respectively, with ϵ_v and ϵ_c as their corresponding eigenvalues. δv is defined self-consistently as[S1]

$$\delta v(\mathbf{r},\omega) = \delta v_{\text{ext}}(\mathbf{r},\omega) + \delta v_H(\mathbf{r},\omega) + \int d^3\mathbf{r}'\,f_{xc}(\mathbf{r},\mathbf{r}',\omega)\delta\rho(\mathbf{r}',\omega)\ , \tag{3}$$

where δv_{ext} and δv_H represent the change in the external and Hartree potentials respectively. f_{xc} is often known as the exchange-correlation kernel[S2], and is given by the second derivative of the exchange-correlation energy with respect to density. Taking the adiabatic approximation[S1,S3], means that the frequency dependence of f_{xc} can be neglected, resulting in

$$f_{xc}(\mathbf{r}, \mathbf{r}') = \frac{\delta^2 E_{xc}[\rho]}{\delta\rho(\mathbf{r})\delta\rho(\mathbf{r}')} \ . \tag{4}$$

With these definitions, it is a simple task to follow the derivation set out in previous work[S3,S4,S5], leading to the Casida formulation of the TDDFT eigenvalue equation:

$$\begin{pmatrix} A & B \\ -B & -A \end{pmatrix} \begin{pmatrix} X \\ Y \end{pmatrix} = \omega \begin{pmatrix} X \\ Y \end{pmatrix} \ . \tag{5}$$

Here, A and B are matrices, that can be written in a basis of the valence and conduction ground-state Kohn-Sham eigenstates as

$$A_{cv,c'v'} = \delta_{c,c'}\delta_{v,v'}(\epsilon_c - \epsilon_v) + Q_{cv,c'v'} \ , \tag{6}$$

and

$$B_{cv,c'v'} = Q_{cv,c'v'} = \iint d^3\mathbf{r}\, d^3\mathbf{r}'\, \psi_c^*(\mathbf{r})\psi_v^*(\mathbf{r}) \left[\frac{1}{|\mathbf{r} - \mathbf{r}'|} + f_{xc}(\mathbf{r}, \mathbf{r}')\right] \psi_{c'}(\mathbf{r}')\psi_{v'}(\mathbf{r}') \ . \tag{7}$$

The two parts of the eigenvector part of Eq. (5), X and Y, represent excitation and de-excitation processes respectively[S5]. The coupling between these two processes can be neglected by assuming that the off-diagonal blocks B are zero (although the contribution of the coupling matrix Q to the on-diagonal blocks A is still included). This is known as the Tamm-Dancoff approximation (TDA)[S6], and has the advantage of reducing the non-Hermitian eigenvalue problem in Eq. (5) to a Hermitian one:

$$AX = \omega X \ . \tag{8}$$

As noted in the main text, the TDA usually gives reliable excitation frequencies, but can give significant errors in some situations, and performs more poorly for the computation of oscillator strengths[S5]. Because of the reduction in complexity, and thus computational cost, the TDA is used throughout this work.

S3 Algorithm for TDDFT calculations in ONETEP

To calculate the N_ω lowest excitation frequencies of the system, the function

$$\Omega = \sum_i^{N_\omega} \omega_i = \sum_i^{N_\omega} \frac{\mathrm{Tr}\left(P_i^{\{1\}\dagger} S^c q_i S^v\right)}{\mathrm{Tr}\left(P_i^{\{1\}\dagger} S^c P_i^{\{1\}} S^v\right)} \tag{9}$$

must be minimised with respect to the response density matrices $P_i^{\{1\}}$, whilst constraining the TDDFT eigenvectors to be orthonormal

$$\mathrm{Tr}\left(P_i^{\{1\}\dagger} S^c P_j^{\{1\}} S^v\right) = \delta_{ij} \ . \tag{10}$$

Here, S^c and S^v are the overlap matrices for the set of conduction NGWFs and valence NGWFs respectively. The response density matrices are the TDDFT eigenvectors (X in Eq. (8)) expressed in terms of the NGWFs:

$$P_i^{\{1\}\alpha\beta} = \sum_{cv} \langle\phi^\alpha|\psi_c\rangle\, X_{cv}^i\, \langle\psi_v|\phi^\beta\rangle \ . \tag{11}$$

q_i represents the action of the TDDFT operator on a trial response density matrix, given by

$$q_i^{\alpha\beta} = \left(K_c H^c P_i^{\{1\}} - P_i^{\{1\}} H^v K_v\right)^{\alpha\beta} + \left(K_c V_{\mathrm{SCF}i}^{\{1\}} K_v\right)^{\alpha\beta} \ , \tag{12}$$

where H^v is the ground-state Hamiltonian, H^c is the projected Hamiltonian used in the conduction NGWF optimisation, and K_v and K_c are the corresponding valence and conduction density kernels. $V_{\mathrm{SCF}i}^{\{1\}}$ is the potential generated by the response density, given by

$$\left(V_{\mathrm{SCF}i}^{\{1\}}\right)_{\alpha\beta} = 2\iint d^3\mathbf{r}d^3\mathbf{r}'\ \phi_\alpha^*(\mathbf{r})\phi_\beta(\mathbf{r})\rho_i^{\{1\}}(\mathbf{r}')\left[\frac{1}{|\mathbf{r}-\mathbf{r}'|} + f_{xc}(\mathbf{r},\mathbf{r}')\right] \ , \tag{13}$$

where $\rho_i^{\{1\}}(\mathbf{r}) = \sum_{cv}\psi_c(\mathbf{r})X_{cv}^i\psi_v(\mathbf{r})$. If a hybrid functional is being used, a contribution from exact exchange should be added to $V_{\mathrm{SCF}i}^{\{1\}}$, given by[S2,S7]

$$\left(V_{\mathrm{SCF}i}^{\{1\}\mathrm{XX}}\right)^{\alpha\beta} = -2f_{\mathrm{XX}}\sum_{\gamma\delta} P_i^{\{1\}\gamma\delta}\,(\alpha\gamma|\delta\beta) \ . \tag{14}$$

In order to conduct a TD-EMFT calculation, this procedure is modified simply by replacing f_{xc} in Eq. (13) with the expression given in Eq. (6) in the main text. If a hybrid functional is being used in the active region, an exact exchange contribution should also be added, but with the exchange restricted to be within the active region only, modifying Eq. (14) to become

$$\left(V_{\mathrm{SCF}i}^{\{1\}\mathrm{XX}}\right)^{\alpha\beta} = -2f_{\mathrm{XX}}\sum_{\gamma\delta\in A} P_i^{\{1\}\gamma\delta}\,(\alpha\gamma|\delta\beta) \ . \tag{15}$$

S4 Higher energy absorption spectra for water-nitrogen dimer

Fig. S1 presents the absorption spectra of the water-nitrogen dimer including the higher energy bright state discussed in the main text, as calculated in vacuum using a variety of different methods – this is directly comparable to Fig.

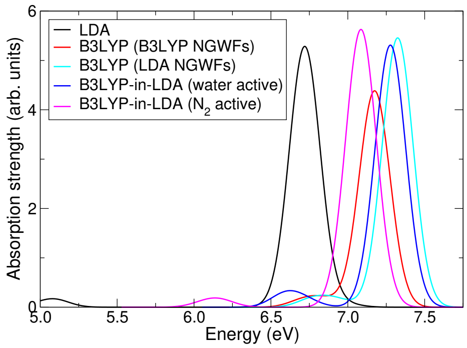

Figure S1: Absorption spectra of the water-nitrogen dimer, including higher energy excitations than those seen in Figs. 2 and 3 in the main text, calculated at various levels of theory: full system LDA (black), full system B3LYP with LDA-optimised NGWFs (cyan), full system B3LYP with B3LYP-optimised NGWFs (red), B3LYP-in-LDA with water as the active region (blue), and B3LYP-in-LDA with nitrogen as the active region (magenta). These results were calculated in vacuum.

2a in the main text. Fig. S2 presents isosurfaces of the response density corresponding to this excitation – this is comparable to Fig. 4 in the main text, although the excitations shown here were computed in vacuum, rather than in implicit solvent. As noted in the main text, LDA does a significantly better job of describing this higher energy excitation relative to B3LYP than excitations of lower energy – the difference between the LDA and full B3LYP excitation energies is 0.45 eV.

The key difference to notice in Fig. S2 compared to Fig. 4 in the main text is that the nitrogen molecule is now more involved in the excitation, particularly in the B3LYP results, although the water molecule is still more dominant. B3LYP and LDA calculations result in somewhat different characters, with the characters of the B3LYP-in-LDA calculations somewhere in between. This enables us to make sense of the results in Fig. S1. The B3LYP-in-LDA result with water as the active region is closer to the full B3LYP result than the B3LYP calculation performed with LDA-optimised NGWFs (the errors are 0.10 and 0.15 eV respectively). This can be understood as the description of the water molecule with B3LYP giving most of the 'correct' result, with the LDA description of the nitrogen molecule providing a contribution that pulls the excitation downwards in energy. The reverse is true for the B3LYP-in-LDA calculation with nitrogen as the active region – compared to the lower energy excitation discussed in the main text, the error for this calculation is somewhat smaller, as the nitrogen molecule is more involved in the excitation, and LDA gives a better description compared to B3LYP.

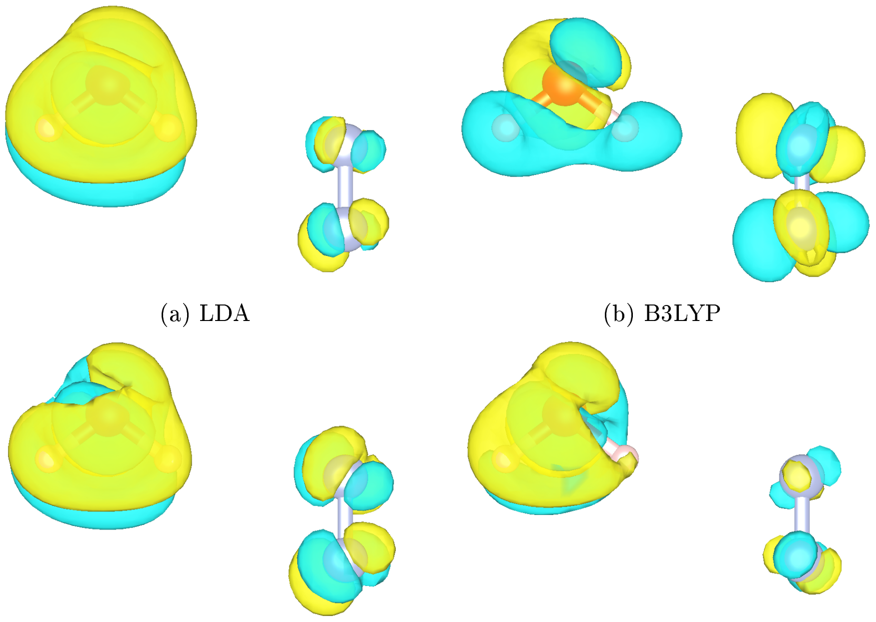

(a) LDA

(b) B3LYP

(c) B3LYP-in-LDA (water active)

(d) B3LYP-in-LDA (N_2 active)

Figure S2: Isosurfaces of the calculated response density for the strongest excitation seen in Fig. S1, calculated at various levels of theory: full system LDA, full system B3LYP (with B3LYP-optimised NGWFs), B3LYP-in-LDA with water as the active region, and B3LYP-in-LDA with nitrogen as the active region. All response densities were computed in vacuum. The isosurfaces are at $|n| = 0.01$ e Å^{-3}, with yellow and blue representing positive and negative response densities respectively. O, N, and H atoms are red, blue, and white respectively. Figures produced using VESTA[S8].

S5 Obtaining explicitly solvated structure for phenolphthalein in water

To obtain the structure shown in Fig. 5b, I used the classical MD code AMBER (v. 16)[S9]. First, I used AMBER to generate a classical force field for the phenolphthalein molecule. This force field was of the GAFF form[S10], and the parameters for it were generated using AMBER's own `antechamber` tool, using the AM1-BCC method[S11] to assign charges. The force field's description of the most important degrees of freedom of the system then needed to be validated against DFT calculations. In this case, I investigated the dihedral rotation of the phenol group, and found that the potential energy surface predicted by the classical force field matched that predicted by DFT up to energies corresponding to 300 K, which was adequate for the purposes of this work.

After obtaining a force field for the phenolphthalein molecule, I solvated it in around 11000 water molecules, described using the TIP3P model[S12]. The energy of this system was then minimised, before being heated from 0 to 300 K over 20 ps. The volume of the system was then allowed to equilibrate in the NPT ensemble at 1 atm and 300 K for 400 ps, before finally performing a production run of 8 ns in the NVT ensemble. The Langevin thermostat was used through, with a collision frequency of 1 ps^{-1}, and a time step of 2 fs was also used throughout. To enable the use of such a relatively large time step, I constrained all bonds involving hydrogen using the SHAKE algorithm[S13,S14]. The snapshot was then extracted from the trajectory obtained in the production run.

S6 Pentacene in *p*-terphenyl absorption spectra

Fig. S3 presents the results of the calculations on the three structures of pentacene in *p*-terphenyl, alongside experimental data[S15,S16]. These are the absorption spectra that correspond to the data shown in Table 1 in Section 3.3 of the main text.

References

S1 F. Ding, T. Tsuchiya, F. R. Manby, and T. F. Miller. Linear-response time-dependent embedded mean-field theory. *J. Chem. Theory Comput.*, 13:4216, 2017.

S2 T. J. Zuehlsdorff, N. D. M. Hine, J. S. Spencer, N. M. Harrison, D. J. Riley, and P. D. Haynes. Linear-scaling time-dependent density-functional theory in the linear response formalism. *J. Chem. Phys.*, 139:064104, 2013.

S3 N. T. Maitra. Perspective: Fundamental aspects of time-dependent density functional theory. *J. Chem. Phys.*, 144:220901, 2016.

S4 M. E. Casida and M. Huix-Rotllant. Progress in time-dependent density-functional theory. *Annu. Rev. Phys. Chem.*, 63:287, 2012.

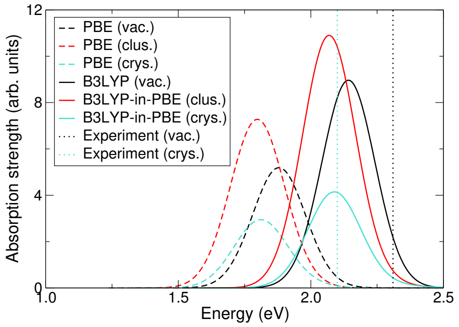

Figure S3: Absorption spectra of pentacene in the three different configurations shown in Fig. 8, as calculated using PBE alone, B3LYP alone, or B3LYP-in-PBE TD-EMFT. Previous experimental data are also shown for reference. Black lines show the spectra calculated for isolated pentacene in vacuum (Fig. 8a in the main text). Red lines show the spectra calculated for pentacene within a cluster of 6 *p*-terphenyl molecules (Fig. 8b in the main text). Turquoise lines show the spectra calculated for pentacene embedded within a *p*-terphenyl crystal, with a periodic cell containing 89 *p*-terphenyl molecules (Fig. 8c in the main text). Dashed lines show the spectra calculated using PBE only. Solid lines show the spectra calculated with B3LYP only (for isolated pentacene in vacuum) or B3LYP-in-PBE TD-EMFT (for the cluster and crystal configurations). The black dotted vertical line marks the experimentally measured energy of the absorption peak for pentacene in vacuum, taken from Ref. S15; the turquoise dotted vertical line marks the same quantity for pentacene embedded within *p*-terphenyl, taken from Ref. S16.

S5 T. J. Zuehlsdorff, N. D. M. Hine, M. C. Payne, and P. D. Haynes. Linear-scaling time-dependent density-functional theory beyond the Tamm-Dancoff approximation: Obtaining efficiency and accuracy with in situ optimised local orbitals. *J. Chem. Phys.*, 143:204107, 2015.

S6 S. Hirata and M. Head-Gordon. Time-dependent density functional theory within the Tamm-Dancoff approximation. *Chem. Phys. Lett.*, 314:291, 1999.

S7 J. Dziedzic, Q. Hill, and C.-K. Skylaris. Linear-scaling calculation of Hartree-Fock exchange energy with non-orthogonal generalised Wannier functions. *J. Chem. Phys.*, 139:214103, 2013.

S8 K. Momma and F. Izumi. VESTA 3 for three-dimensional visualization of crystal, volumetric and morphology data. *J. Appl. Cryst.*, 44:1272, 2011.

S9 R. Salomon-Ferrer, D. A. Case, and R. C. Walker. An overview of the Amber biomolecular simulation package. *Wiley Interdiscip. Rev. Comput. Mol. Sci.*, 3:198, 2013.

S10 J. Wang, R. M. Wolf, J. W. Caldwell, P. A. Kollman, and D. A. Case. Development and testing of a general Amber force field. *J. Comput. Chem.*, 25:1157, 2004.

S11 A. Jakalian, B. L. Bush, D. B. Jack, and C. I. Bayly. Fast, efficient generation of high-quality atomic charges. AM1-BCC model: I. Method. *J. Comput. Chem.*, 21:132, 2000.

S12 P. Mark and L. Nilsson. Structure and Dynamics of the TIP3P, SPC, and SPC/E Water Models at 298 K. *J. Phys. Chem. A*, 105:9954, 2001.

S13 J.-P. Ryckaert, G. Ciccotti, and H. J. C. Berendsen. Numerical integration of the cartesian equations of motion of a system with constraints: molecular dynamics of n-alkanes. *J. Comput. Phys.*, 23:327, 1977.

S14 R. Elber, A. P. Ruymgaart, and B. Hess. SHAKE parallelization. *Eur. Phys. J. Spec. Top.*, 200:211, 2011.

S15 E. Heinecke, D. Hartmann, R. Müller, and A. Hese. Laser spectroscopy of free pentacene molecules (I): The rotational structure of the vibrationless S1←S0 transition. *J. Chem. Phys.*, 109:906, 1998.

S16 J. Köhler, A. C. J. Brouwer, E. J. J. Groenen, and J. Schmidt. On the intersystem crossing of pentacene in p-terphenyl. *Chem. Phys. Lett.*, 250:137, 1996.
